# Increased IL- 36γ in visceral adipose tissue as a key mediator of obesity-driven inflammation in colon cancer

**DOI:** 10.1007/s00109-025-02546-9

**Published:** 2025-04-23

**Authors:** Gema Frühbeck, Sofía Criado, Javier Gómez-Ambrosi, Beatriz Ramírez, Sara Becerril, Amaia Rodríguez, Gabriela Neira, Laura Salmón-Gómez, Víctor Valentí, Rafael Moncada, Jorge Baixauli, Camilo Silva, Victoria Catalán

**Affiliations:** 1 Metabolic Research Laboratory, Cancer Center Clínica Universidad de Navarra (CCUN), Avda. Pío XII, 36, 31008 Pamplona, Spain; 2 CIBER Fisiopatología de La Obesidad y Nutrición (CIBEROBN), Instituto de Salud Carlos III, Pamplona, Spain; 3 Obesity and Adipobiology Group, Instituto de Investigación Sanitaria de Navarra (IdiSNA), Pamplona, Spain; 4 Department of Endocrinology & Nutrition, Cancer Center Clínica Universidad de Navarra (CCUN), Avda. Pío XII, 36, 31008 Pamplona, Spain; 5 Department of Surgery, Cancer Center Clínica Universidad de Navarra (CCUN), Pamplona, Spain; 6 Department of Anesthesia, Clínica Universidad de Navarra, Pamplona, Spain

**Keywords:** Colon cancer, Obesity, Interleukin- 36, Inflammation, Visceral adipose tissue

## Abstract

**Abstract:**

Dysfunctional adipose tissue (AT) in the context of obesity promotes a chronic inflammatory state, associated with worse cancer progression and prognosis. Interleukin (IL)-36γ is a proinflammatory factor increased in obesity. The aim was to analyse the role of IL-36γ in colon cancer (CC) development in patients with obesity. Samples obtained from 74 volunteers (27 with normal weight (NW) and 47 with obesity (OB)) were used in a case–control study. Participants were also subclassified according to the presence of CC (45 without and 29 with CC). HT-29 cells were treated with pro-inflammatory factors, adipocyte conditioned media (ACM) and IL-36γ to evaluate the expression levels of inflammation- and extracellular matrix (ECM) remodelling-related molecules. Increased gene expression levels of *IL36G* and *IL36R* in visceral AT from patients with OB and CC were found. Moreover, mRNA levels of *IL36G* were significantly associated with the gene expression levels of its receptor and relevant genes involved in AT inflammation (*ASC*, *IL1B* and *NLRP6*). Consistently, *IL36G* expression was upregulated by hypoxia, inflammation-related factors (LPS, TNF-α and leptin) and by the adipocyte secretome from patients with obesity in HT-29 cancer cells. Furthermore, we revealed that IL-36γ increased the gene expression levels of inflammation-related genes (*IL36G*, *IL1 A*, *IL1B*, *IL6*, *IL8* and *NGAL*) as well as ECM markers (*MMP9*, *SPP1* and *TNC*) in HT-29 cells. Increased gene expression levels of *IL36G* in VAT from patients with OB and CC may promote a pro-inflammatory microenvironment favourable for tumour progression and migration.

**Key messages:**

- Obesity and colon cancer increase gene expression levels of *IL36G* and *IL36R* in visceral adipose tissue.
- Hypoxia, inflammation-related factors and the adipocyte secretome from patients with obesity upregulate mRNA levels of *IL36G* in HT-29 cancer cells.
- IL-36γ increase the gene expression levels of inflammation-related genes (*IL36G*, *IL1A*, *IL1B*, *IL6*, *IL8* and *NGAL*) as well as ECM markers (*MMP9*, *SPP1* and *TNC*) in HT-29 cells.

**Supplementary Information:**

The online version contains supplementary material available at 10.1007/s00109-025-02546-9.

## Introduction

Obesity prevalence is increasing in the last decades, becoming a key public health challenge [1]. The simplistic energy balance model of obesity has been surpassed by the exposome hypothesis, highlighting the need to consider an integration of multiple factors to fully understand the aetiology and management of this complex disease [2–4]. Importantly, obesity is related with a dysfunction of many other organ systems increasing mortality and triggering high prevalence pathologies including cancer [5, 6]. According to recent studies, obesity promotes the development of thirteen different types of tumours, including colon cancer (CC) [7, 8]. Although the exact molecular mechanisms underlying this association remain unclear [9], the most significant identified causative agents include insulin resistance, increased oestrogen production and dysregulated factors released from dysfunctional adipose tissue (AT) during chronic hypoxia, inflammation and extracellular matrix (ECM) remodelling associated with obesity [9–11]. In fact, hypoxia and inflammation are also hallmarks for cancer development [12].

Among factors released by dysfunctional AT in obesity, the interleukin (IL)−1 family has gained attention for its involvement in modulating both pro- and anti-inflammatory responses that influence tumour progression [13]. IL-36, a relatively new member of the IL-1 family, has emerged as a key player in regulating immune responses, particularly within mucosal tissues such as the gastrointestinal tract [14–16]. The IL-36 family consists of the isoforms IL-36α, IL-36β and IL-36γ, which exert their effects through the IL-36 receptor (IL-36R) as well as the antagonist IL-36Ra, which counteracts the signalling cascade [17]. IL-36 functions as a mediator in the initiation and progression of inflammatory and fibrotic diseases [14, 15, 18]. Although IL-36 has been primarily studied in the context of inflammatory diseases, recent evidence suggests that IL-36 may also influence cancer progression, including CC [19]. The role of IL-36 in CC is complex and multifaceted [20]. On the one hand, IL-36 can enhance immune cell infiltration and promote anti-tumour immunity by activating dendritic cells, macrophages and T cells [21, 22]. On the other, dysregulation of IL-36 signalling may contribute to a pro-tumorigenic environment by sustaining chronic inflammation, which is known to facilitate the development and progression of CC [23–25]. The dual nature of IL-36 as both an immune regulator and a potential promoter of inflammation underscores the need for further investigation into its exact role in CC pathogenesis.

In this context, our group recently described that circulating levels of IL-36γ and its gene expression levels in visceral AT (VAT) were increased in human obesity and obesity-associated type 2 diabetes (T2D) [16]. Other studies had already measured serum IL-36 levels in obesity, finding consistent results [26, 27].

Knowing the role of IL-36 in the obesity-associated low-grade inflammation and the importance of chronic inflammation in the physiopathology of cancer, we hypothesised that increased expression levels of *IL36G* in dysfunctional AT in obesity may favour CC development by increasing inflammation. In this regard, we aimed (i) to analyse circulating concentrations of IL-36γ as well as *IL36G* and *IL36R* gene expression levels in VAT biopsies from patients with and without colorectal cancer subclassified as normal-weight or with obesity, (ii) to examine the role of inflammation-related factors in the expression of *IL36G* and *IL36R* in CC cells, (iii) to study the effect of IL-36γ in the expression of genes related to inflammation and ECM remodelling in CC cells and (iv) to determine the crosstalk between adipocytes and CC cells on the gene expression levels of *IL36G* and *IL36R*.

## Material and methods

### Study population

In this case–control study, 74 subjects (27 with normal-weight (NW) and 47 with obesity (OB)) were included from patients and healthy volunteers attending the Departments of Endocrinology & Nutrition and Surgery at the Clínica Universidad de Navarra. BMI was calculated as weight in kilogrammes divided by the square of height in metres (BMI = weight (kg)/height (m^2^)). The Clínica Universidad de Navarra-Body Adiposity Estimator (CUN-BAE) was used to estimate body fat percentage (BF) [28]. Individuals were subclassified according to BMI as with NW or OB (NW: BMI < 25 kg/m^2^ and OB: BMI ≥ 30 kg/m^2^) as well as according to estimated BF (NW: BF < 20% for males and < 25% for females and OB: BF ≥ 25% for males and ≥ 35% for females). Participants were also classified according to the presence of CC (45 without CC (non-CC) and 29 with CC)) following the established diagnostic protocol for CC. Volunteers with NW and non-CC ones were not given pharmacological treatment, were healthy and had no clinical symptoms or signs of cancer, T2D or liver alteration. All reported investigations were performed conforming to the principles of the Declaration of Helsinki and approved by the Universidad de Navarra Ethical Committee responsible for research (protocol approval number 2018.094). The written informed consent from all volunteers was achieved.

### Surgical procedures and tissue collection

VAT samples were obtained from a subgroup of patients undergoing Nissen fundoplication (for hiatal hernia repair in volunteers with NW, *n* = 11), Roux-en-Y gastric bypass (RYGB) (for severe obesity treatment of patients with OB *n* = 27) and curative resection for primary CC (for CC treatment: *n* = 27, (NW: *n* = 16 and with OB: *n* = 11)). Samples were collected following a laparoscopic procedure at the Clínica Universidad de Navarra. VAT biopsies were taken from different anatomical locations depending on the procedure. For patients undergoing hernia repair, surgery biopsies were obtained from the peritoneal cavity, while for patients undergoing bariatric surgery, samples were taken from the greater omentum. In the case of patients with colon cancer, biopsies were collected from the abdominal cavity, distant from the tumour site. The tissue samples were stored at − 80 °C for further analysis. Since the tissue samples used in this study were obtained from surgical procedures performed on patients, there were occasional instances in which AT could not be collected due to surgical complications.

### Analytical measurements

Plasma and serum samples were obtained by venipuncture after more than 8 h of fasting. Serum glucose, triglycerides and C-reactive protein (CRP) concentrations were analysed by enzymatic spectrophotometric reactions using an automated analyser (Hitachi Modular P800, Roche, Basel, Switzerland), and insulin was measured with an enzyme-amplified chemiluminescence assay (IMMULITE®, Diagnostic Products Corp., Los Angeles, CA, USA) as previously described [29]. Circulating levels of the intestinal damage biomarker calprotectin subunit S100 calcium-binding protein A8 (S100 A8) (DS8900, R&D Systems, Abingdon, UK), IL- 36γ (DY2320 - 05, R&D Systems) lactoferrin (EH309RB, Invitrogen, Carlsbad, CA, USA) and C–C motif chemokine ligand 5 (CCL5) (DRN00B, R&D Systems) concentrations were determined by commercially available ELISA kits following manufacturers’ instructions [29].

### HT- 29 and adipocyte cultures

The HT-29 colorectal adenocarcinoma cell line (HTB™) (ATCC®, Middlesex, UK) was cultured according to the manufacturer’s instructions. Cells were seeded at 3 × 10^5^ cells/well and grown in McCoy’s 5 A medium with L-glutamine (36,600–21, Thermo Fisher Scientific, Waltham, MA, USA) supplemented with 10% foetal bovine serum (N4762, Merck, Darmstadt, Germany) and antibiotic and antimycotic (A5955, Merck) at 37 °C for 24 h. HT- 29 cells were serum-starved for 2 h and then treated for 24 h with adipocyte-conditioned media (ACM) (20% and 40%), cobalt chloride (CoCl_2_) (100 and 200 μM; C8661, Merck) [29, 30], lipopolysaccharide (LPS) (10, 100 and 1000 ng/mL; L2880, Merck) [29–31], lipoteichoic acid (LTA) (10, 100 and 1000 ng/mL; L2515, Merck) [32], leptin (1, 10 and 100 ng/mL; 300–27, Thermo Fisher Scientific) [29, 33], palmitic acid (1, 10 and 100 ng/mL; P0500, Merk) [29] and tumour necrosis factor (TNF)-α (1, 10 and 100 ng/mL; 10,291-TA, R&D Systems) [29, 30, 34]. HT- 29 cells were also treated with increasing concentrations of IL- 36γ (1, 50, 100 and 200 ng/mL; 6835-IL, R&D Systems) [23].

ACM was obtained by the culture and differentiation to mature adipocytes of human stroma-vascular fraction cells (SVFC) isolated from VAT of patients with OB as previously described [29]. ACM was centrifuged and diluted to 20% and 40%.

### RNA isolation and analysis of gene expression levels

Given that VAT contributes to obesity-associated inflammation, mRNA levels of *IL36G* and *IL36R* were analysed in this fat depot. VAT was homogenised using an Ultra-Turrax® T25 basic (IKA-Werke GmbH, Staugen, Germany). The total RNA was isolated using QIAzol® reagent (79,306, Qiagen, Hilden, Germany) for VAT samples and TRIzol® reagent (15,596,018, Invitrogen) for HT-29 cells. The RNeasy Mini kit (74,104, Qiagen) was used to purify samples, and afterwards, they were treated with DNase I (79,254, Qiagen) to remove genomic DNA. In order to synthesise the cDNA, 3 µg of total RNA was reverse-transcribed in a final volume of 60 µL using random hexamers (58,875, Invitrogen) as primers and 300 units of M-MLV reverse transcriptase (28,025–013, Invitrogen) as previously reported [29]. Real-time PCR (7300 Real-time PCR System, Applied Biosystem, Foster City, CA, USA) was performed to determine the transcription levels of collagen (*COL*)*1A1*, *COL6A3*, interleukin (*IL)1A*, *IL1B*, *IL6*, *IL8*, *IL36G*, *IL36R*, lipocalin- 2 (*NGAL*), matrix metalloproteinase 9 (*MMP9*), osteopontin (*SPP1*), transforming growth factor-β (*TGFB*), tenascin C (*TNC*) and vascular endothelial growth factor A (*VEGFA*) as previously reported [29]. Primer Express 2.0 software (Applied Biosystems, Foster City, CA, USA) was used to design primers and probes that were acquired from Genosys (Merck). To avoid genomic DNA amplification, primers or TaqMan® probes were designed to encompass fragments of the areas from the extremes of two exons (Supplemental Table 1). The cDNA was amplified as previously described [29]. The *18S* rRNA (4,310,875, Applied Biosystems) was the endogenous control gene used, and the ΔΔCt formula was applied for relative quantification. Relative gene expression levels were shown as fold expression over the calibrator sample (average of NW individuals without CC or unstimulated HT-29 cells). Negative controls were included in all reactions, and every sample was run in triplicate.

### Statistical analysis

Data are shown as mean ± standard error of the mean (SEM). Because of their non-normal distribution, gene expression levels and CRP concentrations were logarithmically transformed. Two-way analysis of covariance (ANCOVA) was used to study differences between groups adjusted for age. In case of interaction between factors (OB and CC), one-way ANOVA followed by Tukey’s post hoc tests was used to evaluate differences between groups as appropriate. Pearson’s correlation coefficient (*r*) was used to analyse the association between variables. SPSS version 23 (SPSS, Chicago, IL, USA) and GraphPad v9 (San Diego, CA, USA) were used to carry out the calculations.

## Results

### Colon cancer and obesity-increased *IL36G* and *IL36R* mRNA expression in VAT

Table 1 shows the clinical features of the study population. Since patients with CC were significantly older (*P* < 0.001) than volunteers without CC, differences between groups were adjusted by age. Weight, BMI and estimated BF were increased (*P* < 0.001) in patients with OB compared to the NW volunteers. Obesity was associated with higher circulating levels of triglycerides (*P* < 0.01). As expected, CEA levels were increased in patients with CC.

**Table 1:** Anthropometric and biochemical characteristics of the volunteers included in the study

	Non-CC		CC		*P* OB	*P* CC	*P* OB × CC
	NW	OB	NW	OB			
*n*	11 (6, 5)	34 (13, 21)	16 (9, 7)	13 (7, 6)			
Age (years)	44 ± 6.4	46 ± 2.2	60 ± 2.6	64 ± 3.1	0.410	< 0.001	0.652
Weight (kg)	59.5 ± 2.8	115 ± 3.8^***^	64.5 ± 2.2^†††^	80.3 ± 2.9^**, †††, #^	< 0.001	0.010	< 0.001
BMI (kg/m^2^)	20.8 ± 0.5	40.9 ± 1.3^***^	21.8 ± 0.4^†††^	29.5 ± 0.7^***, †††, ###^	< 0.001	0.001	< 0.001
Estimated BF (%)	22.4 ± 2.1	50.0 ± 1.3^***^	28.3 ± 1.6^†††^	35.2 ± 1.7^**, †††^	< 0.001	0.076	< 0.001
Glucose (mg/dL)	88 ± 4.6	119.9 ± 8.4	130.2 ± 14.9	127.5 ± 8.8	0.341	0.217	0.105
Triglycerides (mg/dL)	71 ± 11.1	131.9 ± 9.7	94.2 ± 21.8	182 ± 19	0.005	0.382	0.569
CRP (mg/L)	0.16 ± 0.06	0.81 ± 0.22	1.02 ± 0.65	9.16 ± 0.67^**,††,##^	< 0.001	< 0.001	< 0.001
CEA (ng/mL)	1.58 ± 0.32	1.68 ± 0.28	2.55 ± 0.44	8.41 ± 2.60	0.267	0.021	0.401
Lactoferrin (ng/mL)	38.0 ± 5.8	71.3 ± 7.7	50.9 ± 7.3	55.9 ± 7.6	0.037	0.981	0.197
CCL5 (ng/mL)	5.10 ± 0.73	9.38 ± 0.69^**^	6.68 ± 0.68	5.48 ± 0.72	0.048	0.043	0.002
S100 A8 (ng/mL)	357.4 ± 93.6	552.9 ± 69	233 ± 34.8	387.9 ± 57.4	< 0.001	0.591	0.372

*BMI* body mass index, *BF* body fat, *CC* colon cancer, *CCL5* C–C chemokine ligand 5, *CEA* carcinoembryonic antigen, *CRP* C reactive protein, *NW* normal weight, *OB* obesity, *S100 A8* S100 calcium-binding protein A8. Data are mean ± SEM. Differences between groups were analysed by two-way ANCOVA and one-way ANOVA followed by Tukey’s post hoc tests as appropriate. ^**^P < 0.01 and ^***^P < 0.001 vs NW non-CC; ^††^*P* < 0.01 and ^†††^*P* < 0.001 vs OB non-CC; ^#^*P* < 0.05, ^##^*P* < 0.01 and ^###^*P* < 0.001 vs NW CC

**Fig. 1:**
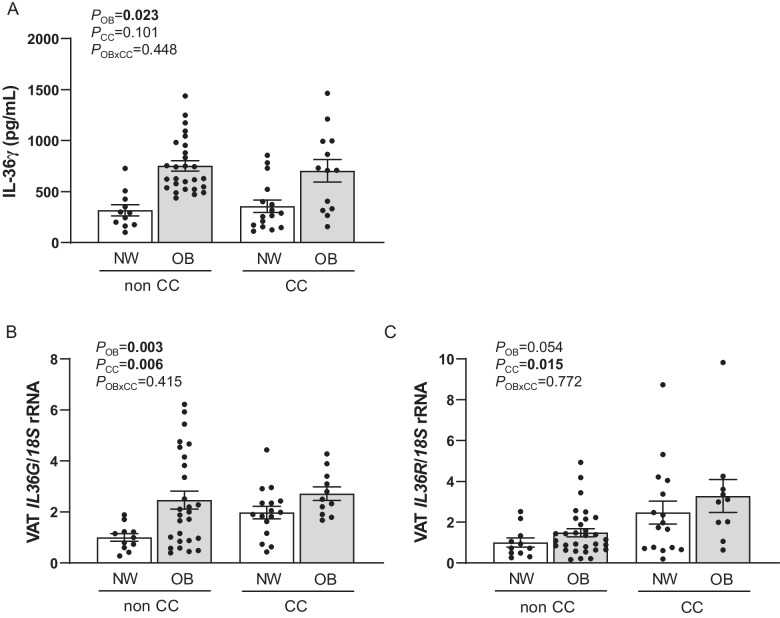
**A** Circulating concentrations of IL- 36γ in normal weight (NW) volunteers and patients with obesity (OB) classified according to the presence or not of colon cancer (CC) (NW-nonCC: *n* = 11; OB-non CC: *n* = 27; NW-CC: *n* = 16; OB-CC: *n* = 13). Gene expression levels of **B** interleukin (*IL*)-*36G* and **C** *IL36R* in visceral adipose tissue (VAT) from normal weight (NW) volunteers and patients with obesity (OB) classified according to the presence or not of colon cancer (CC) (NW-nonCC: *n* = 11; OB-non CC: *n* = 27; NW-CC: *n* = 16; OB-CC: *n* = 11). Differences between groups were analysed by two-way ANCOVA with age as covariable. Bars represent the mean ± SEM

Increased circulating levels of IL-36γ (*P* = 0.023) were found due to obesity, but no differences were observed regarding the presence of colon cancer (Fig. 1A). No sexual dimorphism was found in serum concentrations of IL- 36γ (*P* = 0.287). Since VAT represents a crucial tissue in the obesity-associated chronic inflammation state as well as in colon carcinogenesis, gene expression levels of *IL36G* and *IL36R* in VAT were analysed. Obesity (*P* = 0.003) and CC (*P* = 0.006) increased mRNA levels of *IL36G*. In addition, *IL36R* levels were upregulated (*P* = 0.015) in patients with CC (Fig. 1B)*.* A tendency towards increased levels of *IL36R* due to OB was found, but differences did not reach statistical significance (*P* = 0.054) (Fig. 1C). We also aimed to analyse the association of *IL36G* with relevant factors involved in VAT inflammation (Table 2). Gene expression levels of *NLRP3*, *NLRP6* as well as the effector *IL1B* were significantly increased due to OB and CC. Interestingly, mRNA levels of *IL36G* were positively correlated with *IL36R* (*r* = 0.39,* P* = 0.007), *NLRP6* (*r* = 0.37,* P* = 0.007) and *IL1B* (*r* = 0.31,* P* = 0.035) expression levels.

**Table 2:** Gene expression levels of inflammation-related factors in human visceral adipose tissue

	Non CC		CC		*P* OB	*P* CC	*P* OB × CC
	NW	OB	NW	OB			
*n*	11 (6, 5)	27 (10,17)	16 (9, 7)	11 (6, 5)			
*NLRP1*	1.00 ± 0.03	1.65 ± 0.17	1.21 ± 0.13	1.34 ± 0.21	0.700	0.595	0.624
*NLRP3*	1.00 ± 0.11	15.23 ± 3.03	19.03 ± 4.52	22.41 ± 2.63	0.003	0.022	0.165
*NLRP6*	1.00 ± 0.23	6.94 ± 1.36	6.00 ± 1.63	9.61 ± 2.94	0.006	0.004	0.137
*IL1B*	1.00 ± 0.31	5.71 ± 0.26	5.34 ± 2.04	8.49 ± 2.61	0.016	0.022	0.439
*IL18*	1.00 ± 0.28	0.95 ± 0.12	1.41 ± 0.23	1.14 ± 0.36	0.026	0.004	0.024

*CC* colon cancer, *IL* interleukin, *NLRP* nucleotide-binding oligomerization domain, leucin-rich repeat and pyrin, *NW* normal weight, *OB* obesity. Data are mean ± SEM. Differences between groups were analysed by two-way ANCOVA and one-way ANOVA followed by Tukey’s post hoc tests as appropriate

### Modulation of *IL36G* and its receptor in HT-29 cells under hypoxic and inflammatory conditions

**Fig. 2:**
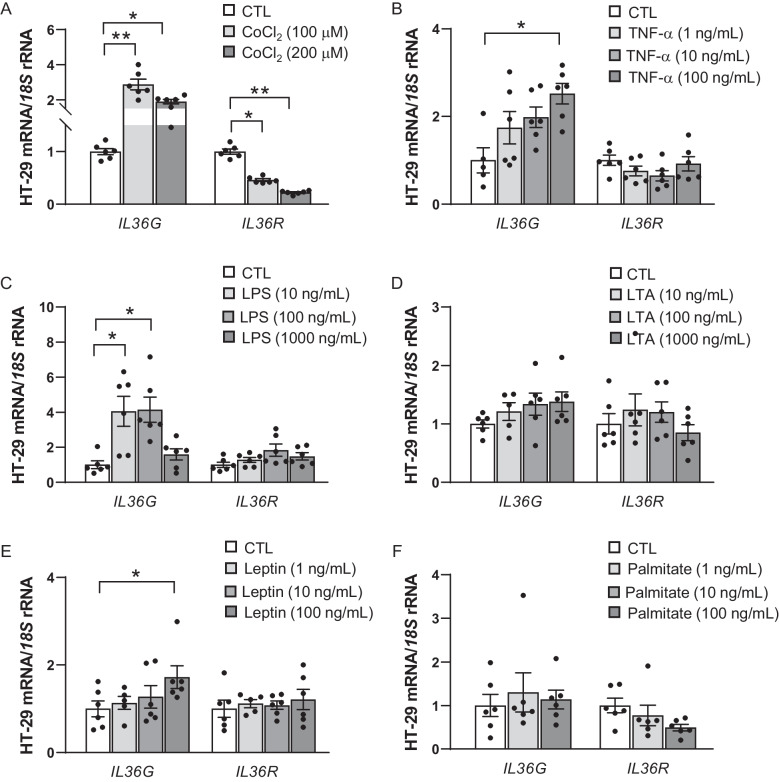
Effect of hypoxia and inflammation-related factors on the gene expression levels of interleukin (*IL*)-*36G* and *IL36R*. HT-29 cells were incubated with **A** cobalt chloride (CoCl_2_), **B** tumour necrosis factor (TNF)-α, **C** lipopolysaccharide (LPS), **D** lipoteichoic acid (LTA), **E** leptin and **F** palmitic acid for 24 h. Values are the mean ± SEM (*n* = 6 per group). Differences between groups were analysed by one-way ANOVA followed by Dunnett’s post hoc tests. ^*^P < 0.05 and ^**^ *P* < 0.01 vs unstimulated cells. CTL, control group

Obesity-induced hypoxia in VAT fosters a pro-inflammatory environment that may contribute to tumour growth and progression by exacerbating inflammatory signalling pathways, including the IL-36/IL-36R axis. After stimulating HT-29 cells with CoCl_2_, a hypoxia-mimicking agent, an increase (*P* < 0.01) in *IL36* expression was observed, while the expression of its receptor, *IL36R*, was significantly decreased (*P* < 0.01) (Fig. 2A). We also investigated the impact of inflammation-related factors altered in obesity on the expression of *IL36G* and *IL36R* in tumour cells. TNF-γ and LPS treatment resulted in a significant increase (*P* < 0.05) in *IL36G* mRNA levels (Fig. 2 B and C) with no changes in *IL36R* expression. Leptin levels are elevated in patients with obesity and, after the stimulation of tumoral cells with this hormone, *IL36G* levels significantly increased (*P* < 0.05) (Fig. 2E). No effects were detected after the stimulation with lipoteichoic acid (LTA) (Fig. 2D) or palmitic acid (Fig. 2F).

### Adipocyte-conditioned media from patients with obesity-induced *IL36G* expression in tumour cells

**Fig. 3:**
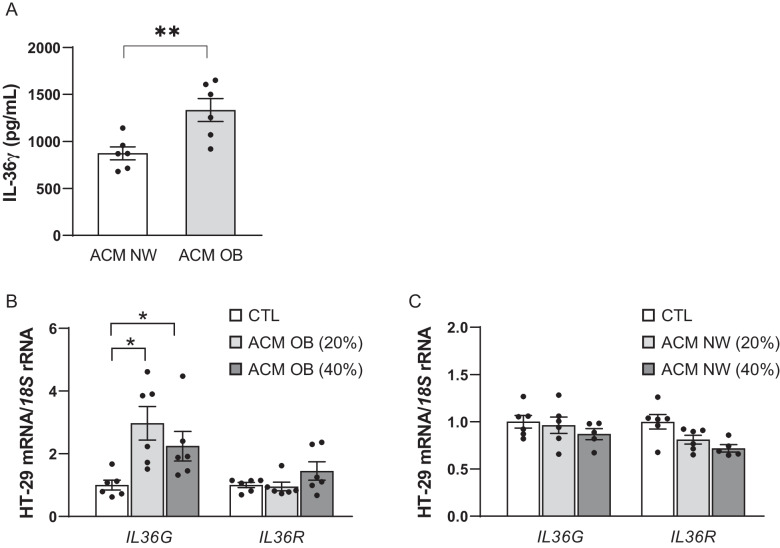
**A** Levels of IL-36γ in the adipocyte-conditioned media (ACM) obtained from individuals with normal weight (NW) and with obesity (OB). Effect of ACM obtained from individuals with **B** OB and with **C** NW on the gene expression levels of interleukin (*IL*)-*36G* and *IL36R* in HT- 29 cells. Bar graphs show the effect of ACM (20 and 40%) from subjects with obesity incubated for 24 h. Values are the mean ± SEM (*n* = 6 per group). Differences between groups were analysed by one-way ANOVA followed by Dunnett’s post hoc tests. ^*^P < 0.05 vs unstimulated cells. CTL, control group

Visceral adipocytes from patients with obesity release different factors that can influence tumour behaviour. For this reason, we herein evaluated the effects of ACM obtained from patients with normal-weight and with obesity on the expression of *IL36G* and *IL36R* in tumour cells. We first assessed the concentrations of IL-36γ in the conditioned media from adipocytes of both groups, finding a significant increase in IL-36γ levels in the ACM obtained from individuals with OB compared to NW patients (*P* = 0.008) (Fig. 3A). We next demonstrated that ACM obtained from patients with OB significantly increased (*P* < 0.05) the expression levels of *IL36G* (Fig. 3B) whereas no differences were observed after the treatment with ACM from individuals with NW (Fig. 3C). These results suggest that the inflammatory mediators present in the ACM in obesity may play a role in modulating the tumour microenvironment, potentially contributing to the progression of CC.

### Impact of IL-36γ on the expression of inflammation- and ECM remodelling-related genes in HT-29 cells

**Fig. 4:**
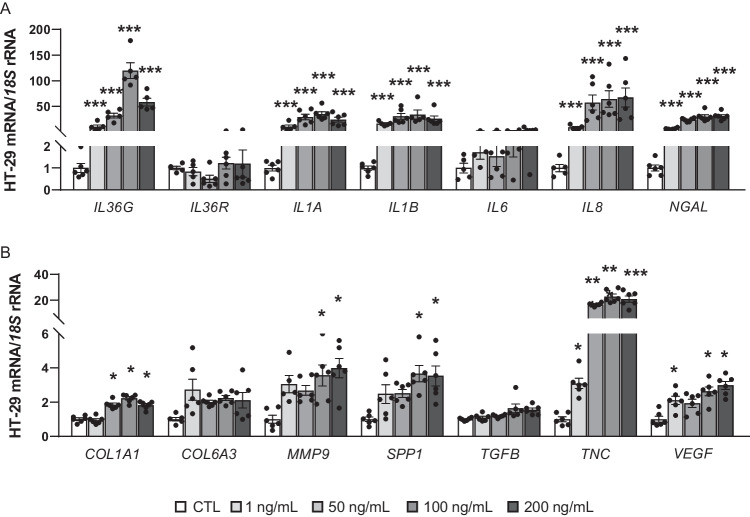
Impact of IL-36γ treatment on the expression levels of **A** pro-inflammatory markers and **B** extracellular matrix proteins in HT- 29 cells. Values are the mean ± SEM (*n* = 6 per group). Differences between groups were analysed by one-way ANOVA followed by Dunnett’s post hoc tests. ^*^P < 0.05, ^**^P < 0.01 and ^***^P < 0.001 vs unstimulated cells*. COL*, collagen; CTL, control group, *IL*, interleukin; *MMP9*, matrix metalloproteinase 9; *NGAL*, lipocalin 2; *SPP1*, osteopontin; *TGFB*, transforming growth factor; *TNC*, tenascin C; *VEGFA*, endothelial growth factor A

HT-29 cells were treated with growing concentrations of IL-36γ to analyse its effect on the expression of genes directly related to inflammation (Fig. 4A). We found that *IL36G* (*P* < 0.001) expression levels were strongly upregulated after IL-36γ treatment while the expression levels of its receptor, *IL36R*, remained unchanged. The inflammatory cytokines *IL1A*, *IL1B* and *IL8* were also upregulated (*P* < 0.001) after IL-36γ treatment. ECM remodelling is particularly relevant in CC development and IL-36 plays a crucial role in modulating the tumour microenvironment. In this context, we detected a significant increase in *NGAL*, *COL1 A1*, *MMP9*, *SPP1*, *TNC* and *VEGF* expression after IL-36 stimulation in HT-29 cells, indicating its potential role in promoting tumour progression through enhanced matrix remodelling (Fig. 4B).

To assess the effects of IL-36γ at physiological levels, we stimulated HT-29 cells with 1 ng/mL of IL-36γ, a concentration chosen based on the mean value measured in our cohort study (886.1 ± 35.9 pg/mL). Importantly, our results showed that gene expression levels of the inflammatory markers *IL1A*, *IL1B*, *IL8* and *IL36* as well as the ECM remodelling-related factors *NGAL*, *TNC* and* VEGF* were significantly increased following a dose-dependent pattern that reinforces the robustness of our findings (Fig. 4 A and B).

## Discussion

IL-36γ exhibits a dual role in inflammation, acting both as a pro-inflammatory mediator and a regulatory factor, therefore being particularly relevant in obesity-related inflammation [19]. Given the established link between chronic inflammation and carcinogenesis, IL-36 may contribute to the cancer response in obesity. In this line, our data provide evidence that (i) obesity and CC increased *IL36G* and *IL36R* mRNA expression in VAT; (ii) hypoxia and the inflammation-related factors LPS, TNF-α and leptin increased gene expression levels of *IL36G* in HT-29 cells; (iii) IL-36γ influenced the expression of key inflammation- and ECM remodelling-related genes in CC cells and (iv) ACM from patients with obesity upregulated the mRNA levels of *IL36G* in HT-29 cells.

Obesity-associated inflammation, especially accompanied by insulin resistance, is a key factor in tumour growth and promotion [35]. Dysfunctional VAT in obesity is known to become a site of chronic inflammation due to the altered lipid storage, increased reactive oxygen species and infiltration of immune cells, such as macrophages, which secrete pro-inflammatory cytokines, including IL-36γ [16, 36]. Many tumour types, including gastric and colon cancer, develop in close contact with adipose tissue, suggesting that dysregulated peritumoural adipocytes may influence the tumour microenvironment by releasing local inflammatory mediators and acting as an energy source, supplying non-esterified fatty acids and growth factors [37–39]. The increased expression of *IL36G* in VAT of patients with obesity and CC may reflect a dysregulated immune response, exacerbating both local and systemic inflammation and contributing to the inflammatory microenvironment surrounding tumours, potentially promoting carcinogenesis. In this context, we previously reported that mRNA levels of *IL36R* were increased in the SVFC compared to adipocytes whereas no differences regarding the expression levels of *IL36G* were found [16]. A significant role of IL-36 in the inflammation of colonic mucosa and in intestinal diseases has been proposed [40, 41]. However, differences in the association between the specific expression of IL-36 isoforms with CC development and survival outcomes exist. Specifically, patients with elevated IL-36γ or reduced IL-36γ levels showed improved survival times in cases of metastatic disease [18, 20]. IL-36 exerts its biological functions by binding to its receptor, IL-36R. The observed upregulation of *IL36R* in VAT from patients with CC, together with the tendency towards increased expression in obesity, supports evidence that the IL-36 axis may be actively involved in the pro-inflammatory signalling within VAT. In this sense, previous in vitro studies revealed that the stimulation of visceral adipocytes with IL-36γ implicates a feedback loop of *IL36G* expression [16]. In the intestine, IL-36R signalling is activated following tissue damage, promoting regeneration and providing protection during episodes of intestinal inflammation and mucosal healing [42]. In this context, a critical role for IL-36γ and IL-36Ra in gut inflammation and tumorigenesis has been demonstrated, proposing that targeting the IL-36γ/IL-36R signalling balance may offer a potential therapeutic strategy for inflammatory bowel disease and gastrointestinal cancers [24]. Previous studies have shown that IL-36 cytokines can activate different inflammasome-related pathways [43, 44]. In the present study, a positive correlation of *IL36G* expression with markers of the inflammasome complex, including *NLRP6*, *ASC* and *IL1B* was detected. This finding suggests that IL-36 might act synergistically with inflammasome activation, reinforcing the concept that IL-36 could be a mediator of inflammation in VAT in the context of obesity and CC.

Hypoxia in VAT, mainly induced by adipocyte hypertrophy and hyperplasia, is another important contributor to AT inflammation, potentially exacerbated by the mechanical stress caused by altered extracellular matrix remodelling and tissue fibrosis [36, 45, 46]. The impact of hypoxia on the tumour microenvironment was also evident in our study. Stimulation of HT-29 cells with CoCl_2_, a hypoxia-mimicking agent, resulted in a significant increase in *IL36G* expression, while the expression of its receptor, *IL36R*, was notably decreased. This phenomenon may indicate a mechanism of receptor desensitisation or adaptation in tumour cells to the hypoxic environment. These findings suggest that hypoxic conditions modulate the IL-36 signalling pathway, potentially contributing to alterations in immune and inflammatory responses in the tumour microenvironment. Understanding these complex regulatory mechanisms is crucial for developing effective therapeutic and preventive strategies. In our study, the selected concentrations of CoCl_2_ are representative to induce moderate hypoxia without causing significant cytotoxic effects, while still promoting hypoxia-related responses at the transcriptional and protein levels. High concentrations of CoCl₂ have been reported to induce cellular stress, which could explain the observed slight downregulation of *IL36G* at the highest concentration tested. Possible mechanisms include oxidative stress, altered signalling pathways or feedback inhibition mechanisms regulating gene expression under hypoxic conditions.

We also investigated the effects of other inflammatory factors altered in obesity on *IL36G* and *IL36R* expression in tumour cells. Reportedly, TLR ligands stimulated the expression of *IL36G* in monocyte-derived macrophages, keratinocytes, hepatocytes and adipocytes [16, 47–49]. In this line, we found that *IL36G* expression levels were significantly increased in HT-29 cells after LPS and TNF-α treatments. According to these results, pathogen-associated molecular patterns (LPS) and damage-associated molecular patterns released from injured epithelial cells (TNF-α) may induce a local inflammatory response in colon adenocarcinoma cells that involve the activation of IL- 36γ. Furthermore, the upregulation of *IL36G* in tumoral cells may also reflect a possible tissue repair function. No differences were observed in *IL36R* expression levels. Of note, we found that low and medium doses of LPS upregulated *IL36G* expression in HT-29 cells, while high concentrations showed no significant impact. A possible explanation is that higher LPS concentrations may induce a form of desensitisation or feedback inhibition in HT-29 cells regarding *IL36G* expression since prolonged or excessive exposure to the inflammatory stimuli lead to tolerance mechanisms, with cell responses diminishing over time to the stimulus [50–52]. Additionally, possible cytotoxic effects at higher concentrations of LPS may impair the ability of cells ability to properly respond, suppressing the expected increase in *IL36G* expression. Furthermore, given its strong pro-inflammatory activity, LPS elicits a broad network of partially redundant regulators to ensure proper modulation of the immune response [53]. This regulatory diversity and functional overlap likely confer adaptability, allowing for precise control of inflammatory gene expression. High levels of leptin, a signature of obesity, have been recognised as a significant risk factor and an important prognostic marker in CC, impacting on cell behaviour including proliferation, apoptosis or migration [54–57]. Interestingly, leptin enhanced the *IL36G* expression in tumour cells in our in vitro study. This relationship suggests that excessive leptin released from AT may promote IL-36 signalling in tumour cells, linking obesity with amplified inflammatory responses that could facilitate cancer progression. The observed concentration-dependent effects in *IL36* expression levels, particularly regarding CoCl_2_ and LPS may result from a combination of receptor saturation, adaptive cellular responses and potential feedback mechanisms. To validate these hypotheses, comprehensive approaches incorporating pathway inhibition assays and gene-editing techniques are warranted. Specifically, blocking key signalling pathways, such as NF-κB and HIF-1α, through pharmacological inhibitors or siRNA-mediated silencing would help delineate their precise contributions to IL- 36 regulation under different experimental conditions. Additionally, CRISPR-based knockdown or overexpression strategies could provide further insights into the role of specific regulatory elements involved in these concentration-dependent effects. Implementing these methodologies would not only clarify the molecular mechanisms underlying our observations but also enhance our understanding of IL-36 modulation in the context of hypoxia and inflammation.

The role of visceral adipocytes as a source of pro-inflammatory factors was further corroborated by our observation that ACM from patients with obesity significantly increased *IL36G* expression in tumour cells. This finding highlights the influence of the VAT microenvironment on tumour behaviour and underscores the importance of understanding how factors released by adipocytes contribute to the inflammatory and metabolic dysregulation seen in obesity-associated CC [58].

Stimulation with IL-36γ led to a significant increase in its own expression in HT-29 cells, while the expression levels of its receptor, *IL36R*, remained unchanged. This self-upregulation suggests a potential autocrine feedback mechanism, where tumoral cells amplify their own inflammatory signalling in response to IL-36γ stimulation. The unchanged expression of *IL36R* may indicate a form of receptor desensitisation, a threshold for receptor expression or a feedback loop wherein tumour cells enhance their inflammatory profile in response to IL-36γ levels downregulating the expression of its receptor. These results highlight a complex regulatory relationship between IL-36γ and its receptor in the tumour microenvironment. The remodelling of the ECM is particularly relevant in the context of CC development, as it facilitates cellular migration, invasion and metastasis. In this sense, the substantial increase in *COL1A1* and other ECM remodelling-related genes, such as *MMP9* and *TNC*, after IL-36γ stimulation in HT- 29 cells underscores the role of this cytokine in modulating the tumour microenvironment. The upregulation of *COL1A1* suggests that IL-36γ plays a pivotal role in creating a supportive environment for tumour progression by altering the composition and structure of the ECM, thereby promoting tumour cell invasion and migration. Previous studies have shown that IL-36γ increases the expression of cell–matrix adhesion genes such as *Col6a1*, *Col1a1* and *Col4a1* in mouse colon organoids mainly through the JNK pathway [24].

The study may be bound to some limitations. Further studies in larger cohorts to improve our understanding of the role of IL-36 in obesity-associated CC are needed. Since the prevalence of colorectal cancer is higher in the elderly population, the average age of our CC group was elevated and, therefore, a study including younger patients would be of great interest. Other potential factors should be taken into account in the in vivo studies, such as CC-related genes or hereditary syndromes as well as in the in vitro studies such as the use of other colon adenocarcinoma cell lines. A remarkable point to consider is that we measured *IL36G* and *IL36R* gene expression in VAT samples from well-characterised patients with obesity and CC. Additionally, the control of confounders and clinically impactful results strengthen its overall significance. Functional assays would provide additional insights into the impact of the crosstalk between AT and cancer cells to further elucidate the biological relevance of the observed transcriptional changes. In addition, the study of the combined effects of both hypoxia and adipocyte-conditioned media will reveal the potential synergistic effects of these factors to better understand their interactive role in modulating inflammation in colorectal cancer. While our in vitro experiments provide valuable insights into the molecular mechanisms underlying IL-36 regulation, they may not fully capture the complexity of the tumour microenvironment and the systemic factors that influence disease progression in vivo. Moreover, the inherent challenges of translating in vitro findings to in vivo models, including the potential differences in cellular behaviour, immune responses and tumour dynamics, highlight the need for further validation. In this regard, the application of more complex in vivo models, including genetically modified mice and patient-derived xenografts, will be critical to better mimic the human tumour microenvironment and more accurately evaluate the therapeutic potential of IL-36 modulation. Furthermore, the use of combination therapies in these models, integrating IL-36-based strategies with existing treatment modalities to assess their synergistic effects, will help for advancing our understanding and the clinical applicability of the findings. These efforts will not only refine our knowledge of the role of IL-36 in obesity-related CC but also contribute to the development of more effective therapeutic approaches for this patient population.

**Fig. 5:**
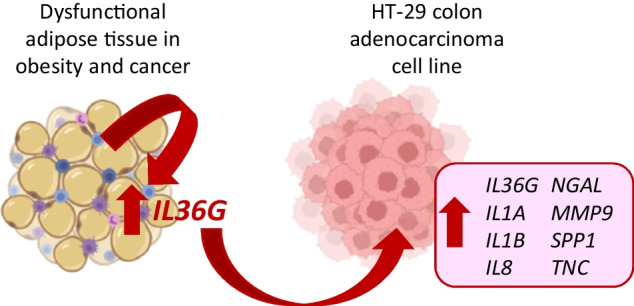
Increased gene expression levels of *IL36G* in VAT of patients with obesity and CC induce a pro-inflammatory microenvironment favourable for tumour progression and migration by promoting its own expression and upregulating crucial inflammation- and ECM-related factors in colon adenocarcinoma cells. *COL*; collagen; *IL*, interleukin; *MMP9*, matrix metalloproteinase 9; *NGAL*, lipocalin 2; *SPP1*, osteopontin; *TNC*, tenascin C

In conclusion, the increased expression of *IL36G* in VAT of patients with obesity and CC underscores its importance in the tumour microenvironment and suggests that the inflammatory milieu and the remodelling of the ECM associated with obesity may drive the upregulation of IL-36 signalling pathways, which are known to contribute to tumorigenesis (Fig. 5). It has to be stressed that IL-36 family members may exhibit a dichotomous nature in inflammation being tissue- and time-specific [15, 18, 20]. Given the chronic inflammatory state associated with both OB and CC, targeting the IL-36 signalling axis could offer a novel therapeutic approach. The ability of IL-36 to influence immune cell recruitment and activation, particularly T cells and macrophages, suggests that modulating its activity may enhance anti-tumour immunity and reduce inflammation-driven tumour progression. In addition, IL- 36 could be strategically integrated with existing therapies, such as anti-inflammatory agents, to improve treatment outcomes in this specific patient group. By regulating IL- 36 expression or signalling, it may be possible to counteract immune evasion mechanisms commonly observed in individuals with OB and CC, thereby improving the effectiveness of current therapeutic strategies. Moreover, IL-36 may also serve as a potential biomarker for identifying patients who could benefit from such combination therapies, ultimately contributing to more personalised and targeted treatment approaches in precision oncology. Future studies should focus on elucidating the underlying molecular mechanisms of IL-36 signalling in the tumour microenvironment, as well as exploring the potential of IL-36 as a biomarker for prognosis and a target for treatment in patients with CC.

## Supplementary Information

Below is the link to the electronic supplementary material.

Supplementary file1 (DOCX 15 KB)

## Data Availability

The datasets used and/or analysed during the current study are available from the corresponding author upon reasonable request.
