# Supplementary material for: Increased IL- 36γ in visceral adipose tissue as a key mediator of obesity-driven inflammation in colon cancer
Source: J Mol Med (Berl). 2025 Apr 23;103(6):699–711. doi: 10.1007/s00109-025-02546-9 (PMC12141383; doi:10.1007/s00109-025-02546-9)
Supplement: Supplementary file 1 — Supplementary file1 (DOCX 15 KB) [file 109_2025_2546_MOESM1_ESM.docx]

**Supplemental Table 1. Sequences of the primers and TaqMan^®^ probes.**

| **Gene (GenBank accession)** | | **Oligonucleotide sequence (5’-3’)** | | | |
| --- | --- | --- | --- | --- | --- |
| *COL1A1* (NM_000088.3) | |  | | | |
| Forward | | CTCCCGGGCCTCAAGGTAT | | | |
| Reverse | | TTGCTCCAGAGGGACCTTGTT | | | |
| TaqMan^®^ Probe | | FAM-TCTTCCTGGCCCCTCTGGTGAACCT-TAMRA | | | |
| *COL6A3* (NM_004369.3) | |  | | | |
| Forward | | GACGGAGATCTGGCTGATTTACA | | | |
| Reverse | | AGATGCATTAGCCGCTCCAA | | | |
| TaqMan^®^ Probe | | FAM-AGAACCTCCGCCAAGAAGGAGTCCGT-TAMRA | | | |
| *IL1A* (NM_000575) | |  | | |  |
| Forward | | GTTCTGAAGAAGAGACGGTTGAGTTT | | |  |
| Reverse | | AAGTTGTATTTCACATTGCTCAGGAA | | |  |
| TaqMan^®^ Probe | | FAM-CATCGCCAATGACTCAGAGGAAGAAATCA-TAMRA | | |  |
| *IL1B* (NM_000576) | |  | | | |
| Forward | | CAGTGGCAATGAGGATGACTTG | | | |
| Reverse | | GTAGTGGTGGTCGGAGATTCGTA | | | |
| TaqMan^®^ Probe | | FAM-TGGCCCTAAACAGATGAAGTGCTCCTTCC-TAMRA | | | |
| *IL6* (NM_000600) | |  | | | |
| Forward | | GCCCTGAGAAAGGAGACATGTAAC | | | |
| Reverse | | ATCCATCTTTTTCAGCCATCTTTG | | | |
| TaqMan^®^ Probe | | FAM-AGGCACTGGCAGAAAACAACCTGAACC-TAMRA | | | |
| *IL8* (NM_000584.3) | |  | | | |
| Forward | | ACCTTTCCACCCCAAATTTATCA | | | |
| Reverse | | TTCTCAGCCCTCTTCAAAAACTTC | | | |
| TaqMan^®^ Probe | | FAM-CCACACTGCGCCAACACAGAAATTATTGTA-TAMRA | | | |
| *IL36* (NM_004530) | |  |  |  |  |
| Forward | | TGTGGGACTTCCACGAAGTG |  |  |  |
| Reverse | | CTTGCTCAAGAGCCTCTGGATAC |  |  |  |
| TaqMan^®^ Probe | | FAM-ACCCCAGTCACTGTTGCTGTTATCACATGC-TAMRA |  |  |  |
| *IL36R* (NM_004530) | |  |  |  |  |
| Forward | | AGATTATGGCCTTCCTTTCATGT |  |  |  |
| Reverse | | GGCGATAAGCCCTCCTATCAA |  |  |  |
| TaqMan^®^ Probe | | FAM-GACCTCCCAGCTCCGGA-TAMRA |  |  |  |
| *MMP9* (NM_004994) | |  | | |  |
| Forward | | GCCCGGACCAAGGATACAGT | | |  |
| Reverse | | CCCCTCAGTGAAGCGGTACA | | |  |
| TaqMan^®^ Probe | | FAM-ACGCGCTGGGCTTAGATCATTCCTCA-TAMRA | | |  |
| *NGAL* (NM_005564) | |  |  |  |  |
| Forward | | CCCAGCCCCACCTCTGA |  |  |  |
| Reverse | | CTTCCCCTGGAATTGGTTGTC |  |  |  |
| TaqMan^®^ Probe | | FAM-CAAGGTCCCTCTGCAGCAGAACTTCCA-TAMRA |  |  |  |
| *SPP1* (NM_000582) | |  | | |  |
| Forward | | CATCCAGTACCCTGATGCTACAGA | | |  |
| Reverse | | GGCCTTGTATGCACCATTCAA | | |  |
| TaqMan^®^ Probe | | FAM-ACATCACCTCACACATGGAAAGCGAGGA-TAMRA | | |  |
| *TGFB* (NM_000660) | |  | |  |  |
| Forward | | GCCCAGCATCTGCAAAGC | |  |  |
| Reverse | | TCCTTGCGGAAGTCAATGTACA | |  |  |
| TaqMan^®^ Probe | | FAM-CACCAACTATTGCTTCAGCTCCACGGA-TAMRA | |  |  |
| *TNC* (NM_002160) | |  | |  |  |
| Forward | | AGGCGATCCCAGACAGTCAGT | |  |  |
| Reverse | | TCCAGCTGACAGTAGCCGAATT | |  |  |
| TaqMan^®^ Probe | | FAM-TCCAGCTGACAGTAGCCGAATT-TAMRA | |  |  |
| *VEGF* (NM_001025250) | |  | | |  |
| Forward | | CAGCACAACAAATGTGAATGCA | | |  |
| Reverse | | ACACGTCTGCGGATCTTGTACA | | |  |
| TaqMan^®^ Probe | | FAM-AATCCCTGTGGGCCTTGCTCAGAGC-TAMRA | | |  |

*COL*, collagen; *IL*, interleukin; *MMP*, matrix metallopeptidase; *NGAL*, lipocalin-2; *SPP1*, osteopontin; *TGFB*, transforming growth factor β; *TNC*, tenascin C, *VEGF*, vascular endothelial growth factor.
